# Association of Neutrophil-to-Lymphocyte Ratio and Monocyte-to-Lymphocyte Ratio With Symptom Severity and Subjective Well-Being in Schizophrenia: A Cross-Sectional Study From Central India

**DOI:** 10.7759/cureus.115769

**Published:** 2026-09-04

**Authors:** Rashmi Meena, Ragini Meena, Jiwan S Meena, Rajendra Nigam, Jaiprakash Agarwal, Ramgopal Baghel

**Affiliations:** 1 Department of Pathology, Gandhi Medical College, Bhopal, IND; 2 Department of Obstetrics and Gynecology, Nandkumar Singh Chouhan Government Medical College, Khandwa, IND; 3 Department of Community Medicine, Gandhi Medical College, Bhopal, IND; 4 Department of Psychiatry, Gandhi Medical College, Bhopal, IND; 5 Department of Dermatology, Gandhi Medical College, Bhopal, IND

**Keywords:** antipsychotic medication, brief psychiatric rating scale (bprs), monocyte-to-lymphocyte ratio (mlr), neutrophil-to-lymphocyte ratio (nlr), schizophrenia, subjective well-being under neuroleptic treatment (swn) scale

## Abstract

Background: Systemic inflammation has increasingly been implicated in schizophrenia. The neutrophil-to-lymphocyte ratio (NLR) and monocyte-to-lymphocyte ratio (MLR), derived from routine complete blood count (CBC) investigations, are inexpensive inflammatory indices, but Indian data relating them to symptom severity and subjective well-being remain limited.

Methods: This hospital-based cross-sectional study included 100 adults with schizophrenia (59 receiving antipsychotic treatment and 41 not currently receiving antipsychotic treatment, comprising newly diagnosed antipsychotic-naïve participants and previously diagnosed participants who had not received antipsychotic treatment for at least six months). CBC was performed in all enrolled participants according to the study investigation protocol. Symptom severity was assessed using the Brief Psychiatric Rating Scale (BPRS), and subjective well-being was assessed using the Subjective Well-being under Neuroleptic Treatment (SWN) scale. Parametric analyses were supplemented by nonparametric sensitivity analyses because NLR and MLR were nonnormally distributed. Treatment-stratified correlations and multivariable linear regression with robust standard errors were performed. An additional sensitivity analysis repeated the principal analyses after excluding the 18 newly diagnosed antipsychotic-naïve participants.

Results: Patients receiving antipsychotic treatment had lower NLR (4.57 ± 1.90 vs. 8.94 ± 5.08) and MLR (0.50 ± 0.17 vs. 0.97 ± 0.50) than those not currently receiving treatment (both p < 0.001); however, the treatment-status groups also differed in illness duration and other baseline characteristics. In the pooled sample, NLR and MLR correlated positively with BPRS (r = 0.736 and 0.791; both p < 0.001). These associations remained significant within both treatment strata. Exploratory interaction analysis indicated that the MLR-BPRS association differed significantly by treatment status (interaction B = 30.40; 95% confidence interval (CI), 14.78-46.03; p < 0.001), whereas the NLR-by-treatment-status interaction was not significant. Pooled negative correlations with SWN did not persist within either treatment stratum. In adjusted models, NLR (B = 1.59; 95% CI, 0.76-2.42; p < 0.001) and MLR (B = 18.76; 95% CI, 11.15-26.37; p < 0.001) remained associated with BPRS after multivariable adjustment. After exclusion of the 18 newly diagnosed antipsychotic-naïve participants (n = 82), between-group differences in NLR and MLR and their associations with BPRS persisted; the MLR-by-treatment-status interaction remained significant but was attenuated (B = 24.82; 95% CI, 5.50-44.15; p = 0.012).

Conclusion: Higher NLR and MLR were associated with greater schizophrenia symptom severity within treatment strata and after multivariable adjustment. Between-group differences according to antipsychotic treatment status should be interpreted cautiously because treatment exposure was observational and the groups differed in baseline clinical characteristics.

## Introduction

Schizophrenia is a chronic psychiatric disorder characterized by disturbances in thought, perception, emotion, and behavior, resulting in substantial disability and impaired quality of life worldwide [1-3]. Although its exact pathophysiology remains incompletely understood, increasing evidence implicates immune dysregulation and systemic inflammation in schizophrenia [4-6].

Globally, schizophrenia affects approximately 0.3%-0.5% of the population and remains one of the leading causes of years lived with disability [7]. In India, the Global Burden of Disease Study estimated a prevalence of approximately 0.3%, highlighting the considerable public health burden of this disorder [8].

Peripheral inflammatory indices derived from routine complete blood counts (CBCs) have attracted attention because they are inexpensive and readily available. Neutrophil-to-lymphocyte ratio (NLR) and monocyte-to-lymphocyte ratio (MLR) are practical indicators of systemic inflammatory activity [9-12]. Meta-analyses have demonstrated elevated NLR and alterations in NLR, MLR, and platelet-to-lymphocyte ratio in schizophrenia and nonaffective psychosis [13,14]. Özdin and Böke reported variation in NLR and MLR across clinical stages [15], while Semiz et al. reported higher NLR in schizophrenia than in healthy controls [16]. Antipsychotic treatment, particularly with second-generation agents, may influence peripheral inflammatory indices through immunomodulatory and metabolic effects; therefore, treatment status may modify the relationship between CBC-derived inflammatory markers and schizophrenia symptom severity.

Symptom severity is commonly assessed using the Brief Psychiatric Rating Scale (BPRS) [17-19], whereas subjective well-being can be evaluated using the Subjective Well-being under Neuroleptic Treatment (SWN) scale [20-22]. Although international evidence regarding hematological inflammatory indices is increasing [14,23], corresponding evidence from Indian patients remains limited.

Therefore, this study evaluated the associations of NLR and MLR with schizophrenia symptom severity and subjective well-being and compared these inflammatory indices between patients receiving and not currently receiving antipsychotic treatment in a tertiary care hospital in Central India.

## Materials and methods

Study design and setting

This hospital-based cross-sectional study was conducted jointly in the Departments of Psychiatry and Pathology at Gandhi Medical College and associated Hamidia Hospital, Bhopal, India, from May 2023 to October 2024. The Institutional Ethics Committee approved the study (Approval No. 155/IEC/2023). Written informed consent was obtained from all participants or their legally authorized representatives.

Study participants

Adults aged 18-65 years diagnosed with schizophrenia according to the International Classification of Diseases, 10th Revision, criteria [24] were consecutively recruited from psychiatry outpatient and inpatient services. Participants were classified according to their antipsychotic treatment status at enrolment. The receiving-treatment group included patients who were receiving regular antipsychotic treatment at enrolment. The not-currently-receiving-treatment group included 1) newly diagnosed patients who had not received antipsychotic treatment and 2) previously diagnosed patients who had not received antipsychotic treatment for at least six months before enrolment. Treatment status was verified through psychiatric evaluation, patient/caregiver interview, and available medical records.

Sample size

The sample size was determined a priori for the planned comparison of inflammatory-marker levels between the two treatment-status groups. A standardized effect size of Cohen’s d = 0.70 was assumed for the planned between-group comparison, with a two-sided alpha of 0.05 and 90% power, yielding a minimum required sample of 86 participants [25]. The final sample was increased to 100. The a priori sample-size calculation was not specifically designed to power multivariable regression or interaction analyses. Accordingly, the adjusted regression models were used to assess robustness to measured confounding, whereas marker-by-treatment-status interaction analyses were considered exploratory and require confirmation in larger samples.

Eligibility assessment

Eligibility was assessed using clinical history, caregiver information, psychiatric assessment, physical examination, and available hospital records and clinically indicated laboratory investigations. Exclusion criteria included severe neurological, autoimmune, cardiovascular, or infectious conditions; pregnancy or lactation; unrelated hematological conditions; and use of anti-inflammatory drugs, corticosteroids, antibiotics, immunosuppressants, or lithium within the preceding four weeks. Systematic screening for asymptomatic inflammatory conditions was not performed.

Data collection

Sociodemographic and clinical information was collected using a structured case-record proforma. Socioeconomic status was classified according to the Modified Kuppuswamy Socioeconomic Scale [26]. For adjusted analysis, any substance use was defined as current smoking, tobacco chewing, or alcohol use.

Clinical assessment

Psychiatric symptom severity was assessed using the 18-item BPRS by a trained postgraduate assessor who received structured training in BPRS administration and scoring under the supervision of a psychiatrist [17-19]. Subjective well-being was assessed using the SWN scale [20-22]. Because SWN was developed for patients receiving antipsychotic treatment, analyses involving participants not currently receiving antipsychotic treatment were treated as secondary and interpreted cautiously.

Laboratory data

CBC was performed in all enrolled participants as part of the study investigation protocol. A venous blood sample was collected in an ethylenediaminetetraacetic acid tube and analyzed in the Department of Pathology using a Zybio Z3 automated hematology analyzer (Zybio Inc., Chongqing, China) in accordance with routine quality-control procedures. Blood samples were generally collected during routine morning laboratory hours; however, the exact collection time was not prospectively standardized. Absolute neutrophil count (ANC), absolute lymphocyte count (ALC), and absolute monocyte count (AMC) were recorded. NLR was calculated as ANC/ALC and MLR as AMC/ALC.

Statistical analysis

Continuous variables were summarized as mean ± standard deviation; medians and interquartile ranges were additionally examined for nonnormal variables. Shapiro-Wilk testing showed marked nonnormality for NLR (W = 0.703, p < 0.001) and MLR (W = 0.752, p < 0.001). Although NLR and MLR were nonnormally distributed, normality of predictor variables is not itself an assumption of ordinary least-squares regression. These variables were therefore retained on their original scales to preserve clinical interpretability, while regression assumptions were evaluated using model diagnostics. Welch’s independent-samples t-test was used for continuous group comparisons and chi-square tests for categorical variables. For the education-by-treatment-status table, in which 20% of expected cell counts were <5, the Fisher-Freeman-Halton exact test was additionally used as a sensitivity analysis. Mann-Whitney U tests were used as sensitivity analyses.

Pearson correlations were retained for comparability with the prespecified analysis, with Spearman correlations as sensitivity analyses. Correlations of NLR/MLR with BPRS and SWN were repeated within treatment strata. NLR/MLR-BPRS associations were the principal focus of the correlation analyses; correlations involving ANC, ALC, AMC, and the SWN analyses were treated as supportive or exploratory and interpreted accordingly.

Separate multivariable linear regression models were fitted for NLR and MLR with BPRS as the dependent variable, adjusting for age, sex, treatment status, duration of illness (categorical), and any substance use. HC3 heteroskedasticity-consistent robust standard errors were used to provide robust confidence intervals (CIs) and p values against potential violations of conventional regression error-variance assumptions. Multicollinearity and heteroscedasticity were assessed using variance inflation factors (VIFs) and Breusch-Pagan tests. As an additional robustness analysis, marker-by-treatment-status interaction terms were evaluated in separate adjusted models to formally assess whether the NLR-BPRS and MLR-BPRS slopes differed by treatment status.

Because all 18 newly diagnosed antipsychotic-naïve participants were in the not-currently-receiving-treatment group, a restricted-sample sensitivity analysis was performed after excluding these participants. The resulting sample comprised 82 previously diagnosed participants (59 receiving antipsychotic treatment and 23 not currently receiving treatment). Principal between-group comparisons, illness-duration distribution, treatment-stratified NLR/MLR-BPRS correlations, and the multivariable and marker-by-treatment-status interaction analyses were repeated. Because the newly diagnosed category was absent from the restricted sample, the 1-3-year illness-duration category was used as the reference category in the multivariable models. To further examine the high explanatory variance of the regression models, a covariate-only model was compared with models additionally containing NLR or MLR, and the incremental change in R² (ΔR²) was calculated. All tests were two-sided, and p < 0.05 was considered statistically significant.

## Results

Participant characteristics

A total of 143 patients were screened, of whom 43 were excluded according to the predefined eligibility criteria, leaving 100 participants for analysis. Of these, 59 were receiving antipsychotic treatment, and 41 were not currently receiving antipsychotic treatment. The mean age of the study population was 48.23 ± 8.61 years, and 73 (73.0%) participants were male. Age, sex, marital status, socioeconomic status, comorbidity, and substance use did not differ significantly between the treatment-status groups. In contrast, residence (p = 0.030), educational status (χ² = 12.05, df = 4, p = 0.017; Fisher-Freeman-Halton exact p = 0.014), and duration of illness (χ² = 32.31, df = 4, p < 0.001) differed significantly between groups. All 18 newly diagnosed participants were in the group not currently receiving antipsychotic treatment (Table 1).

**Table 1:** Sociodemographic and clinical characteristics according to antipsychotic treatment status Values are presented as mean ± SD or number (percentage), as appropriate. Welch's independent-samples t-test was used for age, and Pearson's chi-square test was used for categorical variables. For educational status, the Fisher-Freeman-Halton exact test was additionally performed because 20% of expected cell counts were <5. All tests were two-sided; p < 0.05 was considered statistically significant SD: standard deviation; df: degrees of freedom

Variable	Overall (n = 100)	Receiving antipsychotic treatment (n = 59)	Not currently receiving antipsychotic treatment (n = 41)	Test statistic	df	p value
Age, years, mean ± SD	48.23 ± 8.61	49.41 ± 8.38	46.54 ± 8.77	t = 1.64	83.6	0.105
Gender, n (%)						
Male	73 (73.0%)	43 (72.9%)	30 (73.2%)	χ² = 0.001	1	0.974
Female	27 (27.0%)	16 (27.1%)	11 (26.8%)			
Residence, n (%)						
Rural	52 (52.0%)	36 (61.0%)	16 (39.0%)	χ² = 4.69	1	0.030
Urban	48 (48.0%)	23 (39.0%)	25 (61.0%)			
Educational status, n (%)						
Illiterate	8 (8.0%)	6 (10.2%)	2 (4.9%)	χ² = 12.05	4	0.017, exact p = 0.014
Primary	27 (27.0%)	11 (18.6%)	16 (39.0%)			
High school	27 (27.0%)	15 (25.4%)	12 (29.3%)			
Senior secondary	25 (25.0%)	21 (35.6%)	4 (9.8%)			
College	13 (13.0%)	6 (10.2%)	7 (17.1%)			
Marital status, n (%)						
Married	76 (76.0%)	45 (76.3%)	31 (75.6%)	χ² = 0.50	2	0.780
Separated/divorced	14 (14.0%)	9 (15.3%)	5 (12.2%)			
Widowed	10 (10.0%)	5 (8.5%)	5 (12.2%)			
Socioeconomic status, n (%)						
Lower	15 (15.0%)	9 (15.3%)	6 (14.6%)	χ² = 0.05	3	0.997
Upper lower	25 (25.0%)	15 (25.4%)	10 (24.4%)			
Lower middle	33 (33.0%)	19 (32.2%)	14 (34.1%)			
Upper middle	27 (27.0%)	16 (27.1%)	11 (26.8%)			
Comorbidities, n (%)						
Present	31 (31.0%)	16 (27.1%)	15 (36.6%)	χ² = 1.01	1	0.314
Absent	69 (69.0%)	43 (72.9%)	26 (63.4%)			
Any substance use, n (%)						
Yes	58 (58.0%)	35 (59.3%)	23 (56.1%)	χ² = 0.10	1	0.748
No	42 (42.0%)	24 (40.7%)	18 (43.9%)			
Duration of illness, n (%)						
Newly diagnosed	18 (18.0%)	0 (0.0%)	18 (43.9%)	χ² = 32.31	4	<0.001
1-3 years	23 (23.0%)	15 (25.4%)	8 (19.5%)			
4-6 years	23 (23.0%)	17 (28.8%)	6 (14.6%)			
7-9 years	18 (18.0%)	14 (23.7%)	4 (9.8%)			
≥10 years	18 (18.0%)	13 (22.0%)	5 (12.2%)			

Clinical scores and hematological parameters

Patients receiving antipsychotic treatment had significantly lower BPRS scores than those not currently receiving treatment (50.51 ± 10.58 vs. 78.24 ± 10.57; Welch t = -12.90, p < 0.001), whereas SWN scores were significantly higher (71.39 ± 13.58 vs. 43.10 ± 6.22; Welch t = 14.02, p < 0.001). Significant between-group differences were also observed for ANC, ALC, AMC, NLR, and MLR (all p < 0.001). In particular, mean NLR was 4.57 ± 1.90 in the receiving-treatment group compared with 8.94 ± 5.08 in the group not currently receiving treatment, while mean MLR was 0.50 ± 0.17 and 0.97 ± 0.50, respectively. Because several variables were nonnormally distributed, Mann-Whitney U sensitivity analyses were also performed and confirmed significant between-group differences (all p < 0.001) (Table 2).

**Table 2:** Clinical scores and hematological inflammatory markers according to antipsychotic treatment status Values are presented as mean ± SD and median (IQR). Between-group comparisons were performed using Welch's independent-samples t-test, with Hedges' g reported as the standardized effect size. Mann-Whitney U tests were additionally performed as nonparametric sensitivity analyses. All tests were two-sided; p < 0.05 was considered statistically significant BPRS: Brief Psychiatric Rating Scale; SWN: Subjective Well-being under Neuroleptic Treatment; ANC: absolute neutrophil count; ALC: absolute lymphocyte count; AMC: absolute monocyte count; NLR: neutrophil-to-lymphocyte ratio; MLR: monocyte-to-lymphocyte ratio; SD: standard deviation; IQR: interquartile range; df: degrees of freedom

Parameter	Receiving treatment (mean)	Receiving treatment (SD)	Not receiving treatment (mean)	Not receiving treatment (SD)	Welch t	df	Welch p	Hedges g	Receiving treatment (median)	Receiving treatment (IQR)	Not receiving treatment (median)	Not receiving treatment (IQR)	Mann-Whitney U	Mann-Whitney p
BPRS	50.51	10.58	78.24	10.57	-12.90	86.27	<0.001	-2.60	52.00	40.00-60.00	78.00	71.00-83.00	43.00	<0.001
SWN	71.39	13.58	43.10	6.22	14.02	86.85	<0.001	2.51	73.00	61.00-81.00	42.00	39.00-48.00	2343.00	<0.001
ANC, ×10³/µL	6.02	0.62	7.09	0.59	-8.69	88.77	<0.001	-1.74	6.00	5.60-6.55	7.20	6.60-7.40	271.00	<0.001
ALC, ×10³/µL	1.44	0.33	0.93	0.28	8.27	94.07	<0.001	1.62	1.50	1.25-1.70	1.00	0.70-1.20	2134.50	<0.001
AMC, ×10³/µL	0.66	0.10	0.78	0.11	-5.30	78.56	<0.001	-1.10	0.67	0.59-0.72	0.77	0.71-0.84	529.00	<0.001
NLR	4.57	1.90	8.94	5.08	-5.26	47.85	<0.001	-1.22	4.14	3.32-5.16	7.22	6.08-9.86	235.50	<0.001
MLR	0.50	0.17	0.97	0.50	-5.81	46.64	<0.001	-1.35	0.47	0.37-0.56	0.83	0.64-1.14	264.00	<0.001

Median NLR was 4.14 (interquartile range (IQR), 3.32-5.16) in the receiving-treatment group compared with 7.22 (IQR, 6.08-9.86) in the group not currently receiving treatment, while median MLR was 0.47 (IQR, 0.37-0.56) and 0.83 (IQR, 0.64-1.14), respectively. Mann-Whitney U analyses confirmed significant between-group differences for NLR (U = 235.5, p < 0.001) and MLR (U = 264.0, p < 0.001) (Figure 1).

**Figure 1:**
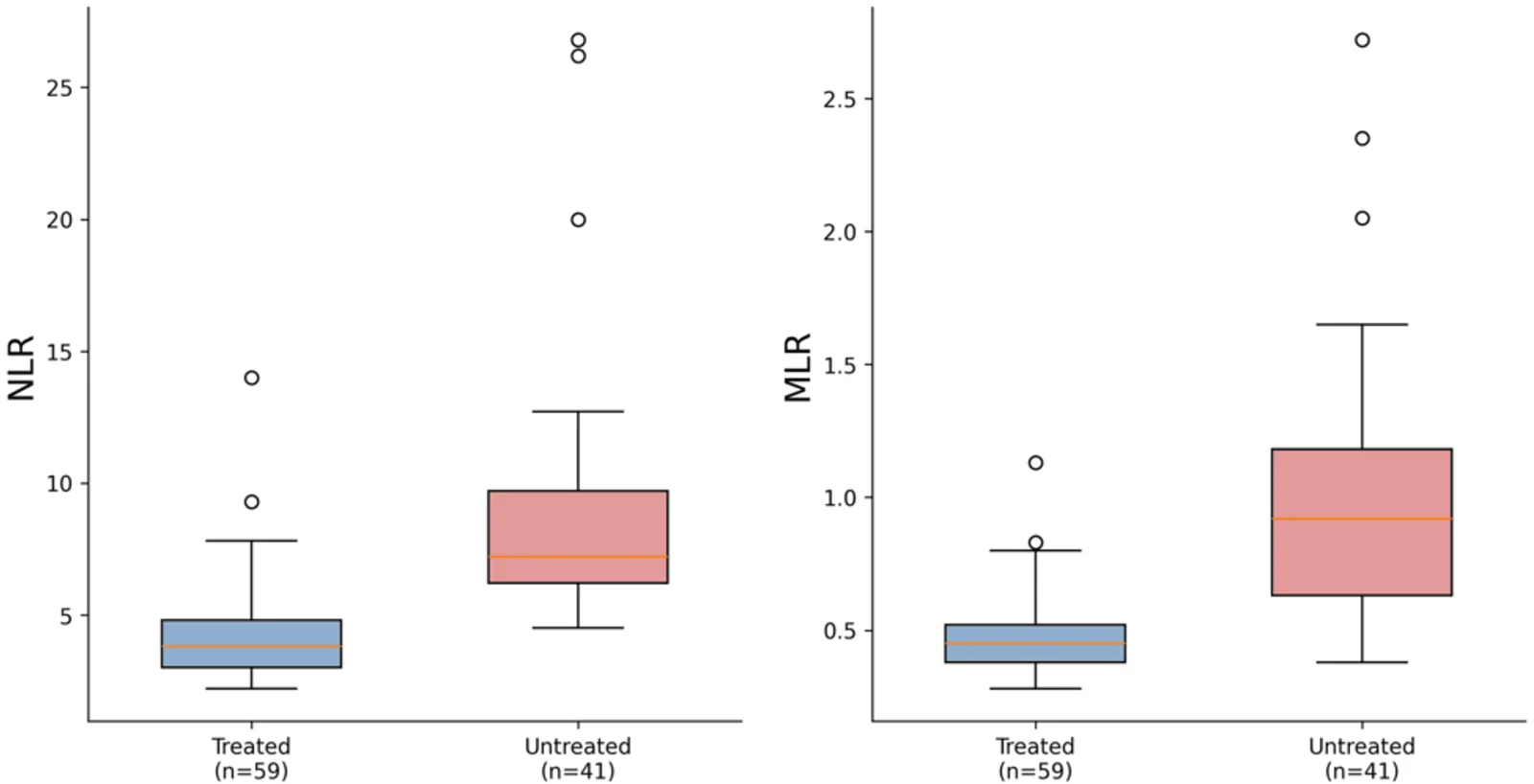
Distribution of NLR and MLR according to antipsychotic treatment status Box plots show the distributions of NLR and MLR among participants receiving antipsychotic treatment (n = 59) and those not currently receiving antipsychotic treatment (n = 41). Boxes represent the interquartile range, horizontal lines indicate medians, whiskers represent the nonoutlier range, and individual points indicate outliers. Between-group comparisons were performed using the Mann-Whitney U test. Both NLR and MLR differed significantly between groups (NLR: U = 235.5, p < 0.001; MLR: U = 264.0, p < 0.001). All tests were two-sided; p<0.05 was considered statistically significant NLR: neutrophil-to-lymphocyte ratio; MLR: monocyte-to-lymphocyte ratio

Correlation analyses

In the overall sample, both NLR and MLR showed strong positive correlations with symptom severity as measured by BPRS (NLR: Pearson r = 0.736, p < 0.001; MLR: r = 0.791, p < 0.001). These associations remained statistically significant after stratification by antipsychotic treatment status. Among participants receiving treatment, the correlations with BPRS were r = 0.627 for NLR and r = 0.726 for MLR, while among those not currently receiving treatment, the corresponding correlations were r = 0.727 and r = 0.801, respectively (all p < 0.001). Spearman's sensitivity analyses supported the overall associations of NLR and MLR with BPRS. In the pooled sample, NLR and MLR were negatively correlated with SWN; however, these associations were not statistically significant within either treatment-status group, suggesting that the pooled associations with subjective well-being were influenced by between-group differences (Table 3).

**Table 3:** Overall and treatment-stratified correlation analyses Pearson correlation coefficients were calculated in the overall sample and separately within treatment-status groups. Spearman rank correlations were additionally calculated in the overall sample as sensitivity analyses. Overall n = 100; receiving treatment n = 59; not currently receiving treatment n = 41. All tests were two-sided; p < 0.05 was considered statistically significant BPRS: Brief Psychiatric Rating Scale; SWN: Subjective Well-being under Neuroleptic Treatment; ANC: absolute neutrophil count; ALC: absolute lymphocyte count; AMC: absolute monocyte count; NLR: neutrophil-to-lymphocyte ratio; MLR: monocyte-to-lymphocyte ratio; r: Pearson correlation coefficient; ρ: Spearman rank correlation coefficient

Marker outcome	Overall Pearson r (p)	Overall Spearman ρ (p)	Receiving treatment Pearson r (p)	Not currently receiving treatment Pearson r (p)
NLR vs. BPRS	0.736 (<0.001)	0.878 (<0.001)	0.627 (<0.001)	0.727 (<0.001)
NLR vs. SWN	-0.396 (<0.001)	-0.525 (<0.001)	-0.073 (0.580)	0.152 (0.343)
MLR vs. BPRS	0.791 (<0.001)	0.887 (<0.001)	0.726 (<0.001)	0.801 (<0.001)
MLR vs. SWN	-0.421 (<0.001)	-0.508 (<0.001)	-0.049 (0.715)	0.158 (0.324)
ANC vs. BPRS	0.806 (<0.001)	0.809 (<0.001)	-	-
ANC vs. SWN	-0.455 (<0.001)	-0.462 (<0.001)	-	-
ALC vs. BPRS	-0.849 (<0.001)	-0.857 (<0.001)	-	-
ALC vs. SWN	0.485 (<0.001)	0.516 (<0.001)	-	-
AMC vs. BPRS	0.634 (<0.001)	0.617 (<0.001)	-	-
AMC vs. SWN	-0.329 (<0.001)	-0.324 (0.001)	-	-

NLR showed a positive association with BPRS scores within both antipsychotic treatment-status groups. The correlation remained statistically significant among participants receiving treatment (r = 0.627, p < 0.001) and those not currently receiving treatment (r = 0.727, p < 0.001). Although the fitted regression lines suggested differences in the NLR-BPRS relationship between the two groups, the adjusted NLR-by-treatment-status interaction was not statistically significant (B = 2.42, 95% CI, -0.60 to 5.43; p = 0.116). Thus, there was insufficient evidence that treatment status significantly modified the association between NLR and symptom severity (Figure 2).

**Figure 2:**
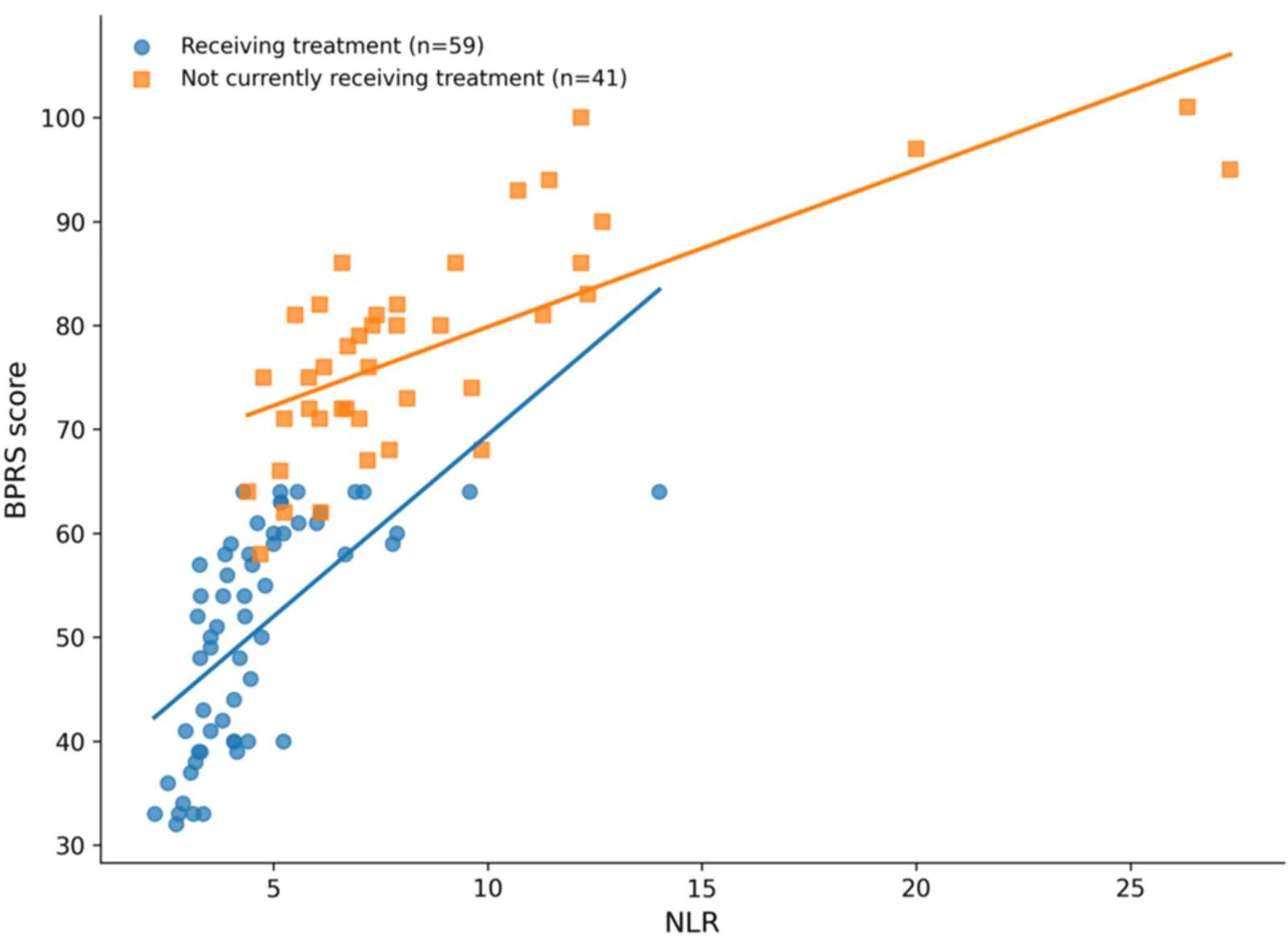
Association between NLR and BPRS score according to antipsychotic treatment status Scatter points represent individual participants, and fitted regression lines show the relationship between NLR and BPRS score within each treatment-status group. The marker-by-treatment-status interaction was evaluated using multivariable linear regression with HC3 robust standard errors. All tests were two-sided; p < 0.05 was considered statistically significant NLR: neutrophil-to-lymphocyte ratio; BPRS: Brief Psychiatric Rating Scale

MLR showed a strong positive association with BPRS scores within both antipsychotic treatment-status groups. The correlation remained statistically significant among participants receiving treatment (r = 0.726, p < 0.001) and those not currently receiving treatment (r = 0.801, p < 0.001). In the adjusted interaction analysis, the MLR-by-treatment-status interaction was statistically significant (B = 30.40; 95% CI, 14.78-46.03; p < 0.001), indicating that treatment status significantly modified the magnitude of the association between MLR and symptom severity (Figure 3).

**Figure 3:**
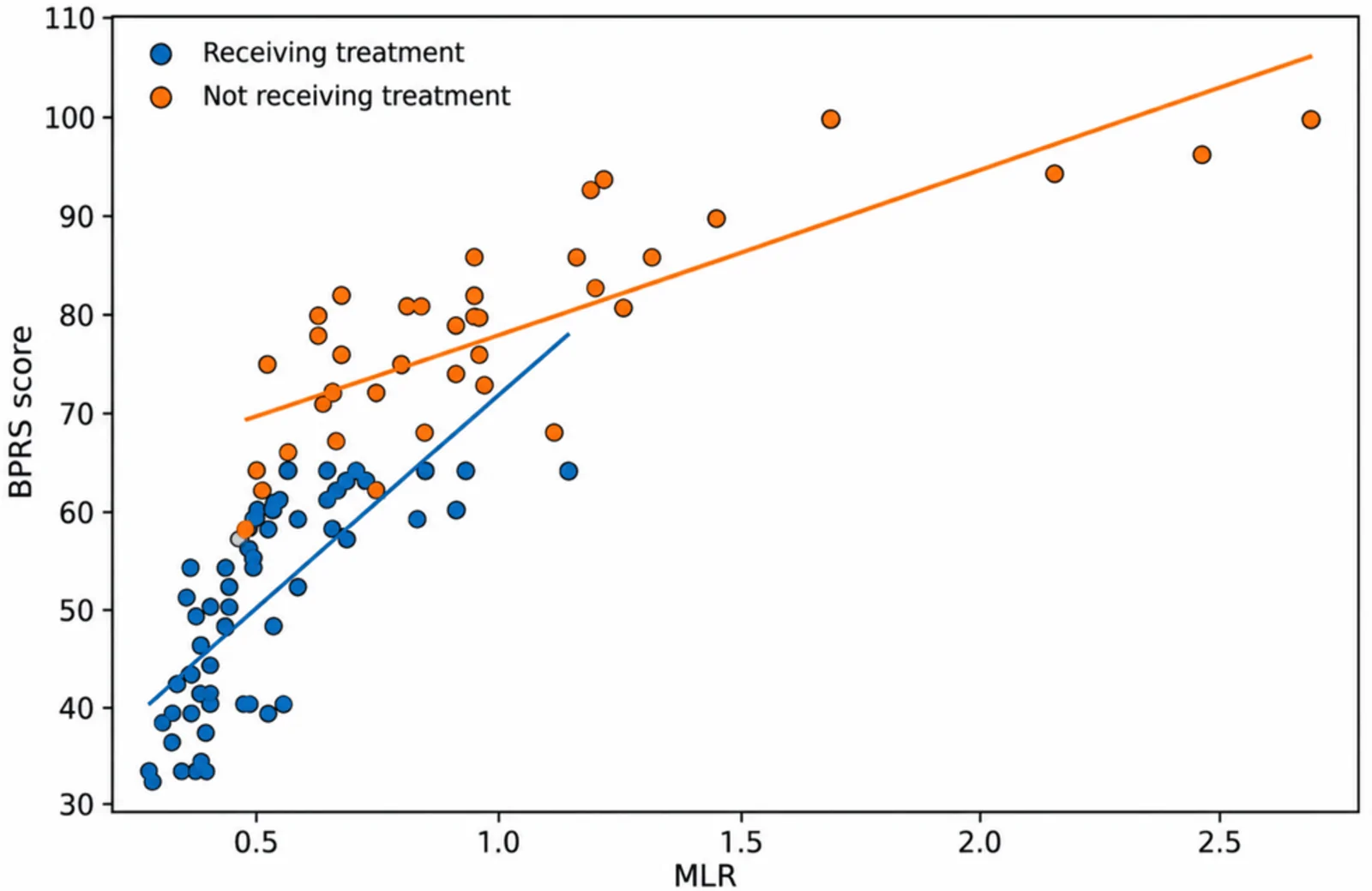
Association between MLR and BPRS score according to antipsychotic treatment status Scatter points represent individual participants, and fitted regression lines show the relationship between MLR and BPRS score within each treatment-status group. The marker-by-treatment-status interaction was evaluated using multivariable linear regression with HC3 robust standard errors. All tests were two-sided; p < 0.05 was considered statistically significant MLR: monocyte-to-lymphocyte ratio; BPRS: Brief Psychiatric Rating Scale

Multivariable regression analyses

In separate multivariable linear regression models adjusted for age, sex, antipsychotic treatment status, duration of illness, and any substance use, both NLR and MLR remained independently associated with BPRS scores. In the NLR model, each one-unit increase in NLR was associated with a 1.59-point increase in BPRS score (B = 1.59; 95% CI, 0.76-2.42; p < 0.001). In the MLR model, each one-unit increase in MLR was associated with an 18.76-point increase in BPRS score (B = 18.76; 95% CI, 11.15-26.37; p < 0.001). The NLR model explained 79.4% of the variance in BPRS scores (R² = 0.794; adjusted R² = 0.773), whereas the MLR model explained 81.7% (R² = 0.817; adjusted R² = 0.798). The maximum VIF was 2.74, indicating no problematic multicollinearity. Breusch-Pagan tests did not indicate significant heteroscedasticity for either model (NLR model, p = 0.421; MLR model, p = 0.258) (Table 4).

**Table 4:** Multivariable linear regression models for BPRS with HC3 robust standard errors Separate multivariable linear regression models were fitted with BPRS score as the dependent variable and either NLR or MLR as the primary inflammatory marker. Both models were adjusted for age, sex, antipsychotic treatment status, duration of illness, and any substance use. HC3 heteroskedasticity-consistent robust standard errors were used. B represents the unstandardized regression coefficient. Reference categories were female sex, not currently receiving antipsychotic treatment, newly diagnosed illness duration, and no substance use. VIF was used to assess multicollinearity. All tests were two-sided, and p < 0.05 was considered statistically significant BPRS: Brief Psychiatric Rating Scale; NLR: neutrophil-to-lymphocyte ratio; MLR: monocyte-to-lymphocyte ratio; B: unstandardized regression coefficient; CI: confidence interval; VIF: variance inflation factor; HC3: heteroskedasticity-consistent covariance estimator

Predictor	NLR model			MLR model		
	B (95% CI)	p value	VIF	B (95% CI)	p value	VIF
Inflammatory marker	1.59 (0.76 to 2.42)	<0.001	1.69	18.76 (11.15 to 26.37)	<0.001	1.87
Age	-0.07 (-0.27 to 0.13)	0.473	1.18	-0.05 (-0.24 to 0.13)	0.564	1.19
Male sex	-4.66 (-11.09 to 1.76)	0.155	2.20	-2.82 (-8.23 to 2.60)	0.308	2.25
Receiving treatment	-18.47 (-22.58 to -14.36)	<0.001	1.63	-17.14 (-21.01 to -13.27)	<0.001	1.69
Duration						
1-3 years	-3.80 (-8.93 to 1.32)	0.146	2.65	-2.53 (-7.26 to 2.21)	0.296	2.69
4-6 years	-4.58 (-10.08 to 0.92)	0.102	2.68	-3.09 (-8.35 to 2.17)	0.250	2.74
7-9 years	-4.08 (-10.20 to 2.04)	0.191	2.58	-2.93 (-8.46 to 2.61)	0.300	2.60
≥10 years	-7.55 (-13.96 to -1.14)	0.021	2.47	-6.44 (-12.30 to -0.57)	0.031	2.49
Any substance use	1.71 (-3.85 to 7.27)	0.546	2.14	1.14 (-3.63 to 5.90)	0.640	2.14

To address the baseline imbalance arising from the inclusion of all 18 newly diagnosed antipsychotic-naïve participants in the not-currently-receiving-treatment group, the principal analyses were repeated after excluding these participants. The restricted sensitivity analysis therefore included 82 previously diagnosed participants, of whom 59 were receiving antipsychotic treatment, and 23 were not currently receiving treatment. After excluding newly diagnosed participants, the distribution of illness duration did not differ significantly between the treatment groups (χ² = 0.864, df = 3, p = 0.834), as shown in Table 5.

**Table 5:** Illness-duration distribution in the restricted sample after exclusion of newly diagnosed antipsychotic-naïve participants (n = 82) Illness-duration distribution was compared using Pearson’s chi-square test (χ² = 0.864, df = 3, p = 0.834). The predefined statistical significance level was p < 0.05 (two-sided)

Duration of illness	Receiving treatment (n = 59)	Not currently receiving treatment (n = 23)
1-3 years	15	8
4-6 years	17	6
7-9 years	14	4
≥10 years	13	5

In the restricted sample, participants receiving antipsychotic treatment continued to have significantly lower BPRS scores than those not currently receiving treatment (50.51 ± 10.58 vs. 72.96 ± 7.01, p < 0.001). NLR (4.57 ± 1.90 vs. 7.03 ± 2.14, p < 0.001) and MLR (0.50 ± 0.17 vs. 0.78 ± 0.22, p < 0.001) also remained significantly lower in the treatment group. Significant between-group differences were also observed for ANC, ALC, and AMC. The SWN difference between treatment groups was also statistically significant; however, this comparison was considered exploratory because SWN was developed primarily in the context of neuroleptic treatment. The complete restricted-sample comparisons are presented in Table 6.

**Table 6:** Clinical scores and hematological parameters after exclusion of newly diagnosed antipsychotic-naïve participants (restricted sample, n = 82) Values are presented as mean ± SD. Between-group comparisons were performed using Welch's independent-samples t-test, with Hedges' g reported as the standardized effect size. The predefined statistical significance level was p < 0.05 (two-sided) ^*^SWN comparisons involving participants not currently receiving antipsychotic treatment were considered exploratory because SWN was developed primarily in the context of neuroleptic treatment BPRS: Brief Psychiatric Rating Scale; SWN: Subjective Well-being under Neuroleptic Treatment; ANC: absolute neutrophil count; ALC: absolute lymphocyte count; AMC: absolute monocyte count; NLR: neutrophil-to-lymphocyte ratio; MLR: monocyte-to-lymphocyte ratio; SD: standard deviation; df: degrees of freedom

Parameter	Receiving treatment, n = 59, mean ± SD	Not currently receiving treatment, n = 23, mean ± SD	Welch t	df	p value	Hedges' g
BPRS	50.51 ± 10.58	72.96 ± 7.01	-11.18	60.39	<0.001	-2.28
SWN^*^	71.39 ± 13.58	43.04 ± 6.51	12.71	76.47	<0.001	2.33
ANC, ×10³/µL	6.02 ± 0.62	6.87 ± 0.57	-5.89	43.50	<0.001	-1.38
ALC, ×10³/µL	1.44 ± 0.33	1.04 ± 0.23	6.19	57.77	<0.001	1.29
AMC, ×10³/µL	0.66 ± 0.10	0.76 ± 0.11	-3.75	36.13	<0.001	-0.97
NLR	4.57 ± 1.90	7.03 ± 2.14	-4.82	36.35	<0.001	-1.24
MLR	0.50 ± 0.17	0.78 ± 0.22	-5.42	32.84	<0.001	-1.48

Both inflammatory ratios remained positively associated with symptom severity in the restricted sample. NLR correlated positively with BPRS overall (r = 0.715, p < 0.001), among participants receiving treatment (r = 0.627, p < 0.001), and among those not currently receiving treatment (r = 0.550, p = 0.007). Similarly, MLR correlated positively with BPRS overall (r = 0.792, p < 0.001), in participants receiving treatment (r = 0.726, p < 0.001), and in those not currently receiving treatment (r = 0.636, p = 0.001). These treatment-stratified correlations are presented in Table 7.

**Table 7:** Correlations of NLR and MLR with BPRS in the restricted sample after exclusion of newly diagnosed antipsychotic-naïve participants (n = 82) Pearson correlation coefficients are presented with corresponding p values. The predefined statistical significance level was p < 0.05 (two-sided) BPRS: Brief Psychiatric Rating Scale; NLR: neutrophil-to-lymphocyte ratio; MLR: monocyte-to-lymphocyte ratio

Association	Overall, n = 82, Pearson’s r (p)	Receiving treatment, n = 59, Pearson’s r (p)	Not currently receiving treatment, n = 23, Pearson’s r (p)
NLR-BPRS	0.715 (<0.001)	0.627 (<0.001)	0.550 (0.007)
MLR-BPRS	0.792 (<0.001)	0.726 (<0.001)	0.636 (0.001)

In the restricted multivariable analyses, NLR remained independently associated with higher BPRS scores (B = 2.93; 95% CI, 1.61-4.25; p < 0.001), as did MLR (B = 34.28; 95% CI, 25.02-43.53; p < 0.001). The NLR model had an R² of 0.713 (adjusted R² = 0.682), whereas the MLR model had an R² of 0.756 (adjusted R² = 0.730). The covariate-only model had an R² of 0.558; the addition of NLR and MLR increased the explained variance by 0.155 and 0.198, respectively. The covariate-only model therefore accounted for a substantial proportion of the explained variance in BPRS.

The NLR-by-treatment-status interaction remained nonsignificant in the restricted sample (B = 2.04; 95% CI, -1.27 to 5.35; p = 0.227). In contrast, the MLR-by-treatment-status interaction remained significant but was attenuated compared with the primary analysis (B = 24.82; 95% CI, 5.50-44.15; p = 0.012). The adjusted MLR-BPRS association was positive in both treatment strata, with a slope of B = 44.16 (95% CI, 29.22-59.10; p < 0.001) among participants receiving treatment and B = 19.34 (95% CI, 6.33-32.34; p = 0.004) among those not currently receiving treatment. Therefore, the interaction reflected a difference in the magnitude rather than the direction of the MLR-BPRS association. Model diagnostics showed no problematic multicollinearity (maximum VIF = 2.38) or evidence of heteroskedasticity (Breusch-Pagan p = 0.839 for the NLR model and p = 0.755 for the MLR model). These multivariable and interaction sensitivity analyses are summarized in Table 8.

**Table 8:** Multivariable regression and marker-by-treatment-status interaction analyses after exclusion of newly diagnosed antipsychotic-naïve participants Multivariable linear regression models were adjusted for age, sex, antipsychotic treatment status, duration of illness, and any substance use, and used HC3 heteroskedasticity-consistent robust standard errors. The one-to-three-year illness-duration category was used as the reference category because newly diagnosed participants were excluded from this restricted analysis. ΔR² represents the increase in explained variance after addition of NLR or MLR to the covariate-only model. Marker-by-treatment-status interaction terms were evaluated in separate adjusted models. The predefined statistical significance level was p < 0.05 (two-sided) BPRS: Brief Psychiatric Rating Scale; NLR: neutrophil-to-lymphocyte ratio; MLR: monocyte-to-lymphocyte ratio; B: unstandardized regression coefficient; CI: confidence interval; VIF: variance inflation factor; HC3: heteroskedasticity-consistent covariance estimator

Analysis	NLR	MLR
Adjusted marker B (95% CI)	2.93 (1.61-4.25)	34.28 (25.02-43.53)
p value	<0.001	<0.001
R²	0.713	0.756
Adjusted R²	0.682	0.730
Covariate-only R²	0.558	0.558
ΔR² after adding marker	0.155	0.198
Maximum VIF	2.38	2.38
Breusch-Pagan p	0.839	0.755
Marker × treatment interaction B (95% CI)	2.04 (-1.27 to 5.35)	24.82 (5.50-44.15)
Interaction p	0.227	0.012
MLR-BPRS slope, receiving treatment, B (95% CI), p	-	44.16 (29.22-59.10); <0.001
MLR-BPRS slope, not currently receiving treatment, B (95% CI), p	-	19.34 (6.33-32.34); 0.004

## Discussion

This hospital-based cross-sectional study demonstrated significant associations of NLR and MLR with schizophrenia symptom severity. Patients receiving antipsychotic treatment had lower NLR, MLR, and BPRS scores than those not currently receiving treatment. However, because the groups also differed in illness duration and other baseline characteristics, these differences cannot be attributed solely to antipsychotic exposure. To address the baseline imbalance arising from the concentration of all 18 newly diagnosed antipsychotic-naïve participants in the group not currently receiving treatment, the principal analyses were repeated after excluding these participants. In the restricted sample of 82 previously diagnosed participants, the distribution of illness duration no longer differed significantly between treatment-status groups (p = 0.834), while significant between-group differences in BPRS, NLR, and MLR persisted. Positive associations between NLR-BPRS and MLR-BPRS also remained evident in both treatment strata. These findings suggest that the principal associations were not solely attributable to the inclusion of newly diagnosed participants in the group not currently receiving treatment. Nevertheless, because treatment exposure was observational and residual measured or unmeasured differences between the groups may remain, these findings should not be interpreted as causal effects of antipsychotic treatment.

The observed associations are consistent with accumulating evidence implicating immune dysregulation and systemic inflammation in schizophrenia [4-6]. Previous studies have reported alterations in peripheral hematological inflammatory indices, including NLR and MLR, in schizophrenia and related psychotic disorders [10-16,23]. Semiz et al. reported elevated NLR in patients with schizophrenia compared with healthy controls [16], while Karageorgiou et al., in a systematic review and meta-analysis, confirmed an overall elevation of NLR in schizophrenia [13]. Mazza et al. demonstrated alterations in both NLR and MLR in nonaffective psychosis [14], supporting the relevance of multiple CBC-derived inflammatory indices. Özdin and Böke reported variation in these indices across different stages of schizophrenia [15], suggesting that illness stage and treatment exposure may influence peripheral inflammatory profiles. Leung et al. further demonstrated an association between NLR and symptom severity in first-episode psychosis [27], consistent with the positive NLR-BPRS association observed in the present study. Our findings extend this literature by demonstrating positive associations of both NLR and MLR with BPRS scores within treatment strata and after adjustment for measured clinical covariates. Differences in absolute inflammatory index values and in the strength of associations across studies may reflect variations in patient characteristics, illness stage, antipsychotic exposure, comorbidity profiles, and laboratory methodology.

Both NLR and MLR showed significant positive correlations with BPRS scores. Importantly, these associations remained significant within both antipsychotic treatment strata and were supported by Spearman sensitivity analyses, reducing the possibility that the pooled correlations resulted solely from between-group separation. These findings are consistent with previous reports linking inflammatory indices with symptom burden in schizophrenia [12,13,23,27,28]. Furthermore, after excluding the 18 newly diagnosed antipsychotic-naïve participants, the positive associations persisted in the restricted sample and across both treatment strata, providing additional evidence that the observed marker-BPRS relationships were not explained solely by the concentration of newly diagnosed participants in one treatment-status group.

Exploratory interaction analyses further clarified these relationships. The NLR-by-treatment-status interaction was not statistically significant in the primary analysis (B = 2.42; 95% CI, -0.60 to 5.43; p = 0.116), indicating insufficient evidence that the NLR-BPRS association differed by treatment status. In contrast, the MLR-by-treatment-status interaction was significant (B = 30.40; 95% CI, 14.78-46.03; p < 0.001). Importantly, this interaction remained significant but was attenuated after exclusion of the 18 newly diagnosed participants (B = 24.82; 95% CI, 5.50-44.15; p = 0.012). In the restricted sample, the adjusted MLR-BPRS association was positive in both treatment strata but was stronger among participants receiving treatment (B = 44.16; 95% CI, 29.22-59.10; p < 0.001) than among those not currently receiving treatment (B = 19.34; 95% CI, 6.33-32.34; p = 0.004). Thus, the interaction reflects a difference in the magnitude rather than the direction of the MLR-BPRS association between treatment strata. One possible explanation is that monocytes and neutrophils are distinct components of innate immunity and may respond differently to the immunomodulatory and metabolic effects of antipsychotic treatment, potentially resulting in differential effects on MLR and NLR. However, this interpretation remains speculative because mechanistic immune pathways and detailed antipsychotic exposure were not assessed in the present study. Accordingly, the association between MLR and symptom severity may vary in magnitude according to treatment status. Given the exploratory nature of the interaction analysis and the smaller not-currently-receiving-treatment subgroup in the restricted sample (n = 23), this finding requires confirmation in larger longitudinal studies with detailed medication-exposure data.

Although NLR and MLR were negatively correlated with SWN scores in the pooled sample, these associations did not persist within either treatment stratum. The pooled relationship with subjective well-being therefore appears to have been substantially influenced by between-group differences. This finding warrants additional caution because SWN was developed in the context of neuroleptic treatment [20-22]. Accordingly, comparisons and associations involving SWN among participants not currently receiving antipsychotic treatment should be regarded as exploratory and should not be interpreted as evidence of a treatment-independent relationship between these inflammatory indices and subjective well-being.

In exploratory analyses, ANC and ALC showed numerically stronger correlations with BPRS than NLR itself, suggesting that individual leukocyte components may contribute substantially to the associations observed for the derived ratio [10-12]. Because these component analyses were supportive and not adjusted for multiple comparisons, they should be interpreted with caution and do not establish the superiority of the individual counts over NLR.

In adjusted regression models, NLR and MLR remained independently associated with BPRS after adjustment for age, sex, treatment status, duration of illness, and any substance use. The restricted sensitivity analysis further clarified the high explanatory variance observed in the primary models. After exclusion of the newly diagnosed participants, R² decreased from 0.794 to 0.713 for the NLR model and from 0.817 to 0.756 for the MLR model. Importantly, the covariate-only model in the restricted sample already explained 55.8% of the variance in BPRS. Addition of NLR increased R² by 0.155, whereas addition of MLR increased R² by 0.198. Thus, the high overall R² should not be interpreted as indicating that either inflammatory marker alone explains approximately 70%-80% of symptom severity. Rather, the explanatory variance reflects the combined contribution of treatment status, illness duration, other included covariates, and the inflammatory marker. The restricted models showed no problematic multicollinearity (maximum VIF = 2.38) or evidence of heteroscedasticity (Breusch-Pagan p = 0.839 for the NLR model and p = 0.755 for the MLR model). Nevertheless, residual or unmeasured confounding and model optimism remain possible, and these findings require confirmation in larger independent cohorts.

NLR and MLR should also be interpreted in light of their biological nonspecificity. Although both are commonly used as peripheral indices of systemic inflammatory status, they may be influenced by a range of clinical, physiological, behavioral, and treatment-related factors. Consequently, the associations observed in this study do not establish that systemic inflammation causes schizophrenia symptoms, nor do they demonstrate that elevated NLR or MLR is specific to schizophrenia. Rather, these markers should be considered nonspecific peripheral inflammatory indices that were associated with symptom severity in the present study. Their potential clinical value, if confirmed, would therefore be as adjunctive markers interpreted alongside clinical assessment rather than as disease-specific biomarkers.

Clinical implications

NLR and MLR can be derived from routinely available CBC reports and may provide complementary information regarding symptom severity, particularly where advanced inflammatory assays are unavailable. However, the present cross-sectional findings do not establish their role in treatment selection or longitudinal monitoring, and these indices should not replace comprehensive psychiatric assessment or established rating scales. Prospective studies incorporating serial measurements of NLR and MLR alongside CRP and other inflammatory biomarkers are needed to determine their potential utility for monitoring symptom severity and treatment response.

Strengths

Strengths include the use of routinely available hematological indices, established clinical assessment scales, treatment-stratified correlation analyses, nonparametric robustness analyses, a restricted-sample sensitivity analysis excluding newly diagnosed antipsychotic-naïve participants, and fully reported multivariable models with robust inference and diagnostic checks.

Limitations

This study has several limitations. Its cross-sectional design precludes causal inference, and the treatment groups differed in illness duration and other baseline characteristics; therefore, observed group differences cannot be attributed solely to antipsychotic treatment. The not-currently-receiving-treatment group included both newly diagnosed antipsychotic-naïve participants and previously diagnosed participants who had not received antipsychotic treatment for at least six months. Although this classification reflected current treatment exposure at enrolment, pooling these clinically distinct subgroups introduced heterogeneity related to illness stage and previous medication exposure. A sensitivity analysis excluding the 18 newly diagnosed antipsychotic-naïve participants reduced this source of heterogeneity and showed persistence of the principal associations; however, the resulting restricted sample was smaller (n = 82), particularly the not-currently-receiving-treatment subgroup (n = 23). Therefore, estimates from the restricted analyses, especially the marker-by-treatment-status interaction estimates, should be interpreted cautiously.

Although treatment status and illness duration were included in the adjusted models without problematic multicollinearity, residual confounding related to these differences remains possible. As participants were recruited from the outpatient and inpatient services of a single tertiary-care hospital, selection and referral bias may limit the generalizability of the findings to community-based populations with schizophrenia.

Although blood samples were generally collected during morning laboratory hours, the exact timing of collection was not prospectively standardized; therefore, some residual variability related to diurnal changes in leukocyte counts cannot be excluded. Systematic screening for asymptomatic inflammatory conditions was not performed. NLR and MLR are nonspecific indices of systemic inflammation and may be influenced by inflammatory comorbidities; therefore, elevations in these indices cannot be attributed specifically to schizophrenia-related inflammation without considering alternative clinical causes. CRP and other inflammatory biomarkers were not systematically measured and therefore could not be evaluated alongside NLR and MLR; future studies incorporating these markers may provide a more comprehensive assessment of systemic inflammation and help determine the independent clinical relevance of CBC-derived indices. Detailed information on antipsychotic type, dose, duration, cumulative exposure, and time since last exposure was unavailable. Potential confounding from BMI, metabolic status, diet, physical activity, smoking intensity, and psychosocial stress was also not fully assessed.

The SWN was developed in the context of neuroleptic treatment [20-22], making its interpretation among participants not currently receiving antipsychotic treatment exploratory. The absence of a healthy control group limits conclusions regarding diagnostic specificity and prevents direct comparison of inflammatory indices with individuals without schizophrenia. Finally, several supportive and exploratory analyses were performed without multiplicity adjustment and should therefore be interpreted cautiously.

## Conclusions

Higher NLR and MLR were consistently associated with greater schizophrenia symptom severity, with these associations persisting within treatment strata, after multivariable adjustment, and in the restricted sensitivity analysis excluding newly diagnosed antipsychotic-naïve participants. The MLR-BPRS association differed significantly by treatment status, whereas no significant interaction was identified for NLR. In contrast, pooled associations with subjective well-being did not persist within treatment strata. Treatment-status comparisons remained observational and should not be interpreted causally.

NLR and MLR may have potential as inexpensive adjunctive inflammatory markers associated with clinical severity, particularly in resource-limited settings, but they should not be considered standalone diagnostic, prognostic, or monitoring biomarkers. Prospective longitudinal studies with standardized laboratory assessment, detailed medication exposure, healthy comparison groups, and additional inflammatory biomarkers are required.
